# The Evaluation of Developmental Dysplasia of Hip from Pediatric Ultrasound Images by Key Point-Based Deep Learning

**DOI:** 10.3390/diagnostics16213649

**Published:** 2026-11-04

**Authors:** Chia-Ho Chuang, Qi-Fang You, Chih-Kai Hong, Chii-Jeng Lin, Mao-Lin Hung, Yung-Nien Sun, Chien-An Shih, Ming-Huwi Horng

**Affiliations:** 1 Department of Computer Science and Information Engineering, National Cheng Kung University, Tainan 701, Taiwan; 2 Department of Orthopedics, National Cheng Kung University Hospital, College of Medicine, National Cheng Kung University, Tainan 704, Taiwan

**Keywords:** pediatric hip ultrasound, developmental dysplasia of the hip (DDH), convolutional neural network, DDH-KPNet, transformer, key point detection

## Abstract

**Background**: Developmental dysplasia of the hip (DDH) is a common hip disorder in infants. Ultrasound is the primary imaging modality for infants younger than 6 months, but measurements using the Graf and Harcke methods depend on examiner experience. This study aimed to develop and internally validate a key point-based deep learning model for the automated measurement of the Graf α and β angles and femoral head coverage. **Methods**: A total of 300 standard-plane hip ultrasound images from 165 infants aged 0–6 months were retrospectively collected at a single tertiary center, and reference key points were determined by consensus of pediatric orthopedic surgeons. We developed a dual-branch network, named “DDH-KPNet,” which combines a UNet++ convolutional branch and a transformer branch fused with attention modules. Performance was evaluated with fivefold cross-validation at three levels: accuracy of key point localization; intraclass correlation coefficient (ICC); and DDH classification. **Results**: The experimental results demonstrated that Graf-based classification achieved accuracy = 0.903, sensitivity = 0.81, and specificity = 0.9497 (under 95% conference interval); Harcke classification achieved accuracy = 0.942, sensitivity = 0.888, and specificity = 0.968 (under 95% conference interval); and ICCs for α = 0.832, β = 0.728, and coverage = 0.923. **Conclusions**: The proposed model showed promising agreement with expert measurements under internal validation. Multicenter external validation and independent diagnostic analysis by different physicians are required before clinical implementation.

## 1. Introduction

Developmental dysplasia of the hip (DDH) is the most common hip disorder in infants, with an incidence rate of approximately 0.1% to 3% [1,2,3]. Most infants do not exhibit discomfort in the early stages, making detection difficult. If not diagnosed and treated in time, it can lead to hip dysplasia, subluxation, and dislocation due to mechanical instability. Physical examination has been the primary method for early screening; however, this method can only detect severe cases of hip dysplasia. Even experienced physicians find it challenging to make a definitive diagnosis in the early stages of the disease. X-rays are not effective for newborns due to the lack of bone ossification, which prevents clear visualization of the hip joint; there are also safety concerns regarding radiation exposure.

**Figure 1:**
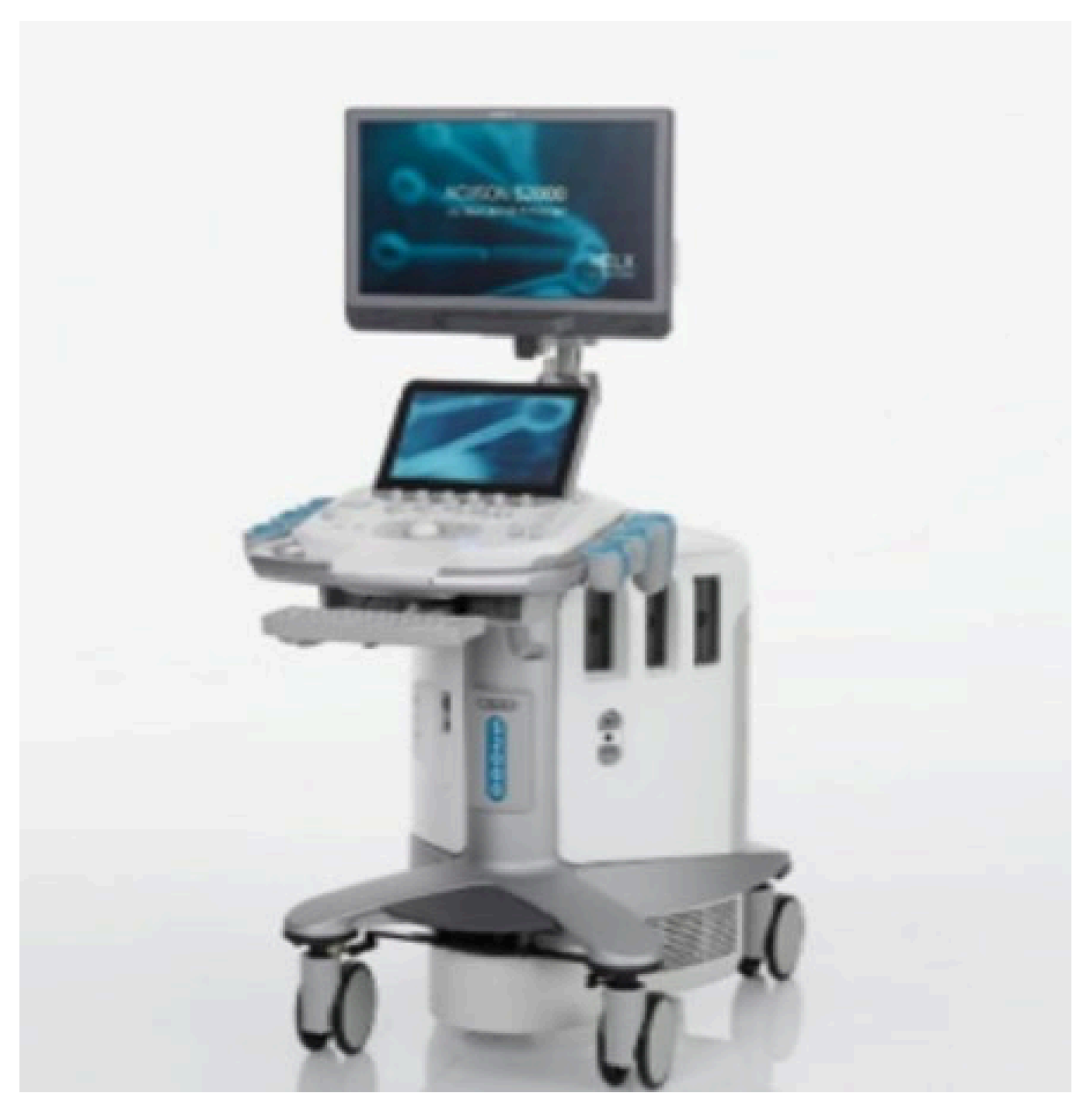
The ultrasound machine used for its acquisition.

Currently, hip ultrasound examination is considered the best screening method (see Figure 1). Its advantages include the absence of radiation and invasiveness, fast examination speed, and no sedation required. Ultrasound allows observation of the hip joint structure and measurement of angles. In the evaluation of DDH using ultrasound, the Graf method [4] is primarily used to measure the α and β angles, while the Harcke method [5] is used to measure femoral head coverage. Although the Graf and Harcke methods are widely used in clinical practice, inconsistencies can easily arise during hip ultrasound screening, with accuracy highly dependent on the equipment used and the skill and experience of the operator. A potential solution to this issue could lie in ultrasound measurements based on deep learning methods, which can ensure more consistent results by eliminating human error.

The neonatal hip ultrasound examination included the neonatal hip ultrasound measurement images and ultrasound machine, as shown in Figure 1, which displays pediatric hip ultrasound images and the acquisition device used for capturing the ultrasound. This method provides orthopedic doctors with real-time imaging of the hip joint, enabling them to quickly and effectively obtain cross-sectional views of the hip structure during clinical diagnosis, as well as measure the positions of various parts of the hip joint. However, the downside of ultrasound is its relatively low resolution, and the image quality is closely related to the angle at which the ultrasound probe is positioned, leading to potential issues of image distortion.

Nowadays, convolutional neural network (CNN) architectures are widely used and continue to dominate in medical image processing. By leveraging convolution operations, CNNs can effectively and rapidly extract local features due to their characteristics of local perception and parameter sharing. However, this also leads to the blurring of positional information in the input data due to compression, resulting in the loss of precise positional information from the original image. To mitigate this loss, the transformer model [6], introduced with the self-attention mechanism, can effectively capture long-distance dependencies, thus enabling global feature extraction while maintaining positional relevance. However, compared with CNNs, the transformer model is less efficient in extracting local features.

Intraobserver and interobserver variabilities [7,8,9] in measuring the α and β angles in a clinical hip ultrasound have been topics of discussion for many years. A recent study [10] indicated that accurate interpretation of ultrasound images depends on performing the screening according to the correct measurement methods. To reduce human error and improve measurement accuracy, various deep learning methods have been proposed, as deep learning techniques have become increasingly prevalent. In previous research, El-Hariri et al. [11] used image segmentation methods, employing U-Net [12] to predict segmentation maps of the hip joint, assisting doctors in reducing the time required to interpret hip images. Hareendranathan et al. [13] used AlexNet [14] to segment hip structures and measured the angles between approximate lines on the segmentation maps to calculate the α and β angles. Stamper et al. [15] improved the U-Net model to segment the femoral head and used an algorithm to locate the bone position to calculate femoral head coverage.

Key point detection is a method that leverages deep learning techniques to identify important feature points in images, such as facial landmarks, object contours, or skeletal joint points. In the field of computer vision, CNNs are widely applied to key point detection. Through multiple layers of convolutional operations, CNNs can effectively learn feature representations from images and predict the coordinates of key points in subsequent feature heatmaps. For developmental dysplasia of the hip (DDH), Chen et al. [16] utilized Unet++ [17] to detect key points by incorporating multiscale feature fusion spatial information, and Lee et al. [18] employed mask R-CNN [19] to segment the hip joint region, and then predicted key points on the hip joint segmentation map. As deep learning technologies advance, research has increasingly focused on enhancing the accuracy and efficiency of key point detection. Techniques such as multiscale feature fusion can improve detection accuracy by considering information from different scales within the image. Additionally, self-attention mechanisms have been introduced into key point detection to help models better focus on important image regions. Hsu et al. [20] used an attention-enhanced YOLOv11 model for automated classification. Shimizu et al. [21] used HigherHRNet-W48 to diagnose DDH and achieved an area under the curve (AUC) of 0.92. Tasolar et al. [22] used DeepLabv3+ segmentation with multiple backbone architectures to determine Graf angles and reported a segmentation accuracy of 94.64%.

**Figure 2:**
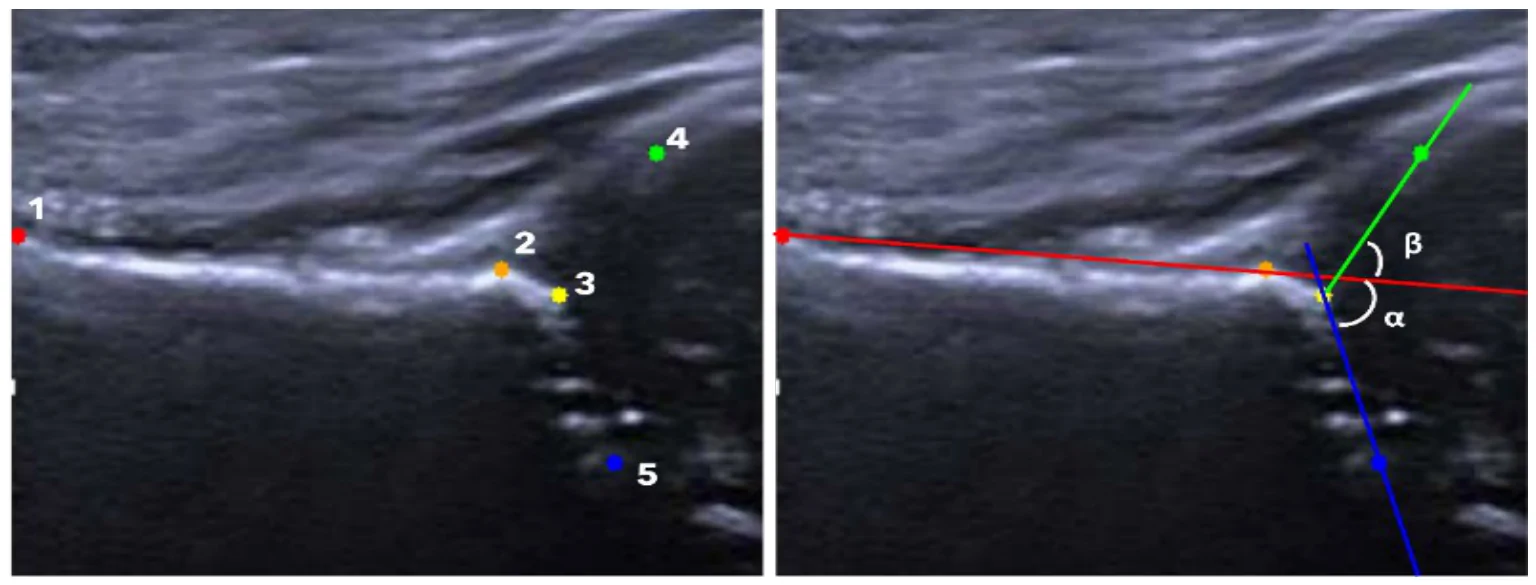
Key point annotation for α and β and Graf method measurement standards. (**Left**) The five key points: (1) cranial point of the straight iliac contour; (2) caudal end of the straight iliac contour; (3) bony rim of the acetabular roof; (4) center of the acetabular labrum; and (5) lower limb of the os ilium. (**Right**) The baseline (red) passes through Points 1 and 2, the bony roof line (blue) through Points 3 and 5, and the cartilage roof line (green) through Points 3 and 4. The α angle is formed by the baseline and the bony roof line, and the β angle by the baseline and the cartilage roof line.

After predicting the key points, we drew three measurement lines: the baseline, drawn through the cranial point (Point 1) and the caudal end (Point 2) of the straight iliac contour; the bony acetabulum line, drawn from the lower limb of the os ilium (Point 5) through the bony rim (Point 3); and the cartilaginous acetabulum line, drawn from the bony rim (Point 3) through the center of the acetabular labrum (Point 4) (Figure 2). The angle between the baseline and the bony acetabulum line is defined as the α angle, and the angle between the baseline and the cartilaginous acetabulum line is defined as the β angle. A larger α angle indicates a more mature hip joint and is the primary criterion for determining the presence of developmental dysplasia of the hip. The β angle is an auxiliary indicator, with a smaller value suggesting a mature hip joint.

For disease classification, as shown in Figure 2 and Table 1, the Graf method classifies hips with an α angle ≥ 60° as Type I (mature). Hips with an α angle of 50–59° are classified as Type IIa (physiologically immature, age < 12 weeks) or Type IIb (delayed ossification, age ≥ 12 weeks); this distinction depends on the infant’s age, rather than on image features [23]. Hips with an α angle of 43–49° and a β angle ≤ 77° are classified as Type IIc (critical range). In this study, Type I hips were considered normal, whereas Type II hips (IIa, IIb, and IIc) were considered abnormal.

**Figure 3:**
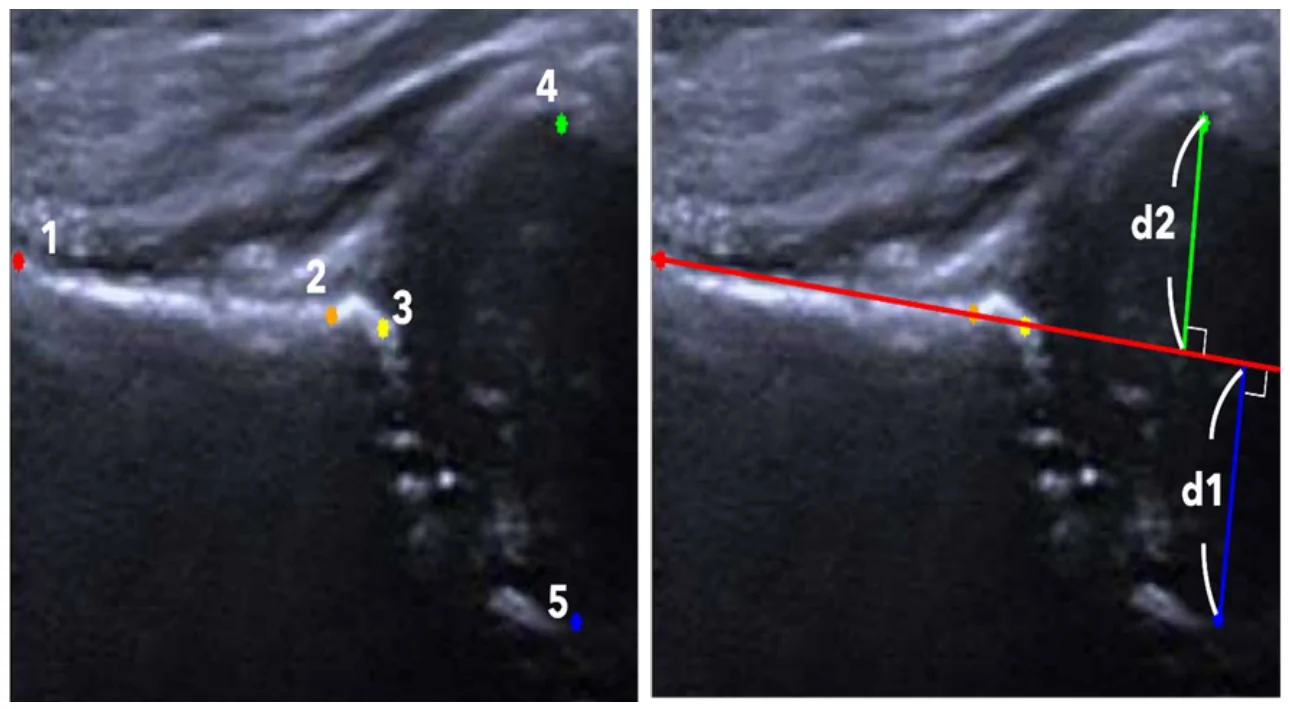
Key point annotation for femoral head coverage and Harcke method measurement standards. (**Left**) The five key points: (1) cranial point of the straight iliac contour; (2) caudal end of the straight iliac contour; (3) bony rim of the acetabular roof; (4) lateral (upper) margin of the femoral head; (5) medial (lower) margin of the femoral head. (**Right**) The iliac plane segment (red) is defined by Points 1–3; d1 and d2 are the perpendicular distances from Points 5 and 4, respectively, to the iliac plane segment.

The Harcke method is another clinical standard for diagnosing DDH, as shown in Figure 3. The key points required for the Harcke method include the cranial point of the straight iliac contour (Point 1), the caudal end of the straight iliac contour (Point 2), the bony rim of the acetabular roof (Point 3), the lateral (upper) margin of the femoral head (Point 4), and the medial (lower) margin of the femoral head (Point 5). The model needs to detect these key points and calculate the femoral head coverage ratio based on their positions.

After predicting the key points, we first measure the iliac plane segment. We define the right end of the iliac plane segment as the line segment connecting the starting point of the iliac linear portion’s right-side elevation with the midpoint of the iliac linear portion’s left side, and the right side of the iliac linear portion forms the iliac plane segment. Then, by projecting the medial (lower) margin of the femoral head (Point 5) onto the iliac plane segment (length d1) and the lateral (upper) margin of the femoral head (Point 4) onto the iliac plane segment (length d2), we can calculate the coverage ratio. The femoral head coverage ratio is calculated as shown in Equation (1). In clinical diagnosis, the threshold for coverage ratio is 50%. A coverage ratio greater than 50% is considered healthy, while less than 50% indicates hip dysplasia (see Figure 3). When d1 is longer than d2, the coverage ratio is greater than 50%, indicating that the femoral head is considered to have normal development if the projection of the medial (lower) margin of the femoral head onto the iliac plane segment is longer.

(1) $$\mathrm{Coverage}=\frac{d1}{d1+d2}$$

**Figure 4:**
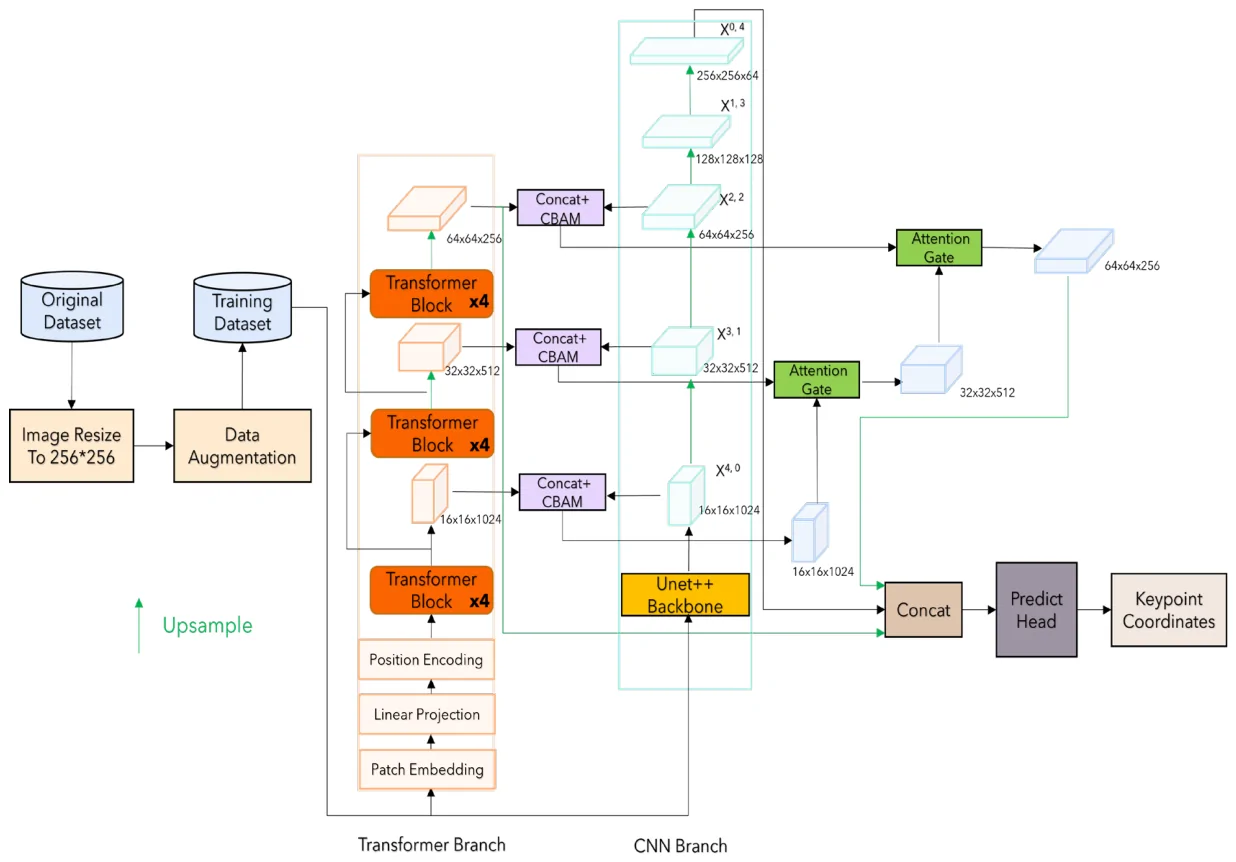
Proposed DDH-KPNet model.

A new model combined the characteristics of CNNs and transformers to propose a key point detection model, called “TransFuse” [24], which has a CNN branch and transformer branch that parallelly transmit features, with an attention module introduced to interactively fuse the multiscale feature maps derived from both branches, thereby improving the accuracy of key point prediction. This paper proposed a new key point detection model, DDH key point detection network (DDH-KPNet), to measure the parameters used for the ultra-sonographic assessment of DDH. The overall architecture of DDH-KPNet model is illustrated in Figure 4. The DDH-KPNet model consists of a dual-branch structure: one branch is a CNN branch using Unet++ as the backbone; the other is a transformer branch based on the self-attention mechanism. Compared with the original TransFuse and previous key point-based DDH studies [16,18,20,21,22], the main modifications and contributions are as follows: (1) the ResNet backbone of the CNN branch was replaced with UNet++; (2) the vision transformer branch was modified to output hierarchical feature maps after every four transformer blocks; (3) the two branches were fused with CBAM, and multiscale features were aggregated with attention gates; (4) key point coordinates were obtained with a DSNT-based prediction head trained with a combined Euclidean distance and Jensen–Shannon divergence loss; and (5) a single framework provides the Graf angles and femoral head coverage from the same image, and key point localization, measurement agreement, and classification are evaluated separately.

## 2. Materials and Methods

### 2.1. Materials

This retrospective study was approved by the Institutional Review Board of National Cheng Kung University Hospital (Approval No.: B-ER-111-022); the requirement for informed consent was waived because of the retrospective design. Infants aged 0–6 months who underwent hip ultrasonography between October 2022 and January 2024 were eligible. By design, Graf types D, III, and IV (decentered) hips were excluded, as were images that were not in the Graf standard plane, showed probe tilt, or had motion artifacts or insufficient quality to identify the required landmarks. The final data set comprised 300 images from 165 infants (87 infants were boys and 78 infants were girls), with normal-to-abnormal ratio of 2:1; 135 infants contributed images of bilateral hips, and the other 30 infants contributed a one-side hip image. Each hip was treated as the unit of analysis. From a clinical perspective, the right and left hips of the same infant are distinct anatomical units whose development frequently differs. Consequently, the two hips of the same infant often differ in sonographic morphology and Graf type, and in clinical practice, each hip is examined, classified, and managed separately [1]. Because the measurement task in this study—the α and β angles and femoral head coverage—is performed on an individual hip, the contralateral hip of the same infant represents a different anatomical structure with its own developmental status, rather than a duplicate of the same hip. In total, 300 infants’ ultrasonic hip images were collected. The median age at examination was 63.2 ± 24.6 days. The DDH-KPNet model had 45.67 M parameters, a training time of about 6.52 h for 240 training samples of each fold, and an inference time pf about 3.415 s; it was developed for this project and written in Python, utilizing the PyTorch framework. The hardware setup included an NVidia GTX 4080 Ti graphics card and an Intel(R) Core (TM) i7-2600 CPU complemented by 8 GB of primary memory, operating in a Linux environment. The floating-point operations (FLOPs) were about 54.312 G.

All examinations were performed by a single examiner with more than 10 years of experience in infant hip ultrasonography, using a single ultrasound system (ACUSON S2000, Siemens Healthineers, Erlangen, Germany) with a 12L4 linear-array transducer (4.0–12.0 MHz). Examinations followed the standard Graf technique: the infant was placed in the lateral decubitus position with the hip in slight flexion (approximately 20°) and slight internal rotation; and the transducer was held parallel to the long axis of the body to obtain a coronal image. Only images in the Graf standard plane—displaying the lower limb of the os ilium, a straight iliac contour at the mid-portion of the acetabular roof, and the acetabular labrum—were retained. The Graf angles and femoral head coverage were measured on the same standard-plane coronal image. The original images measured 1024 × 768 pixels with a pixel spacing of 0.071 mm/pixel.

**Figure 5:**
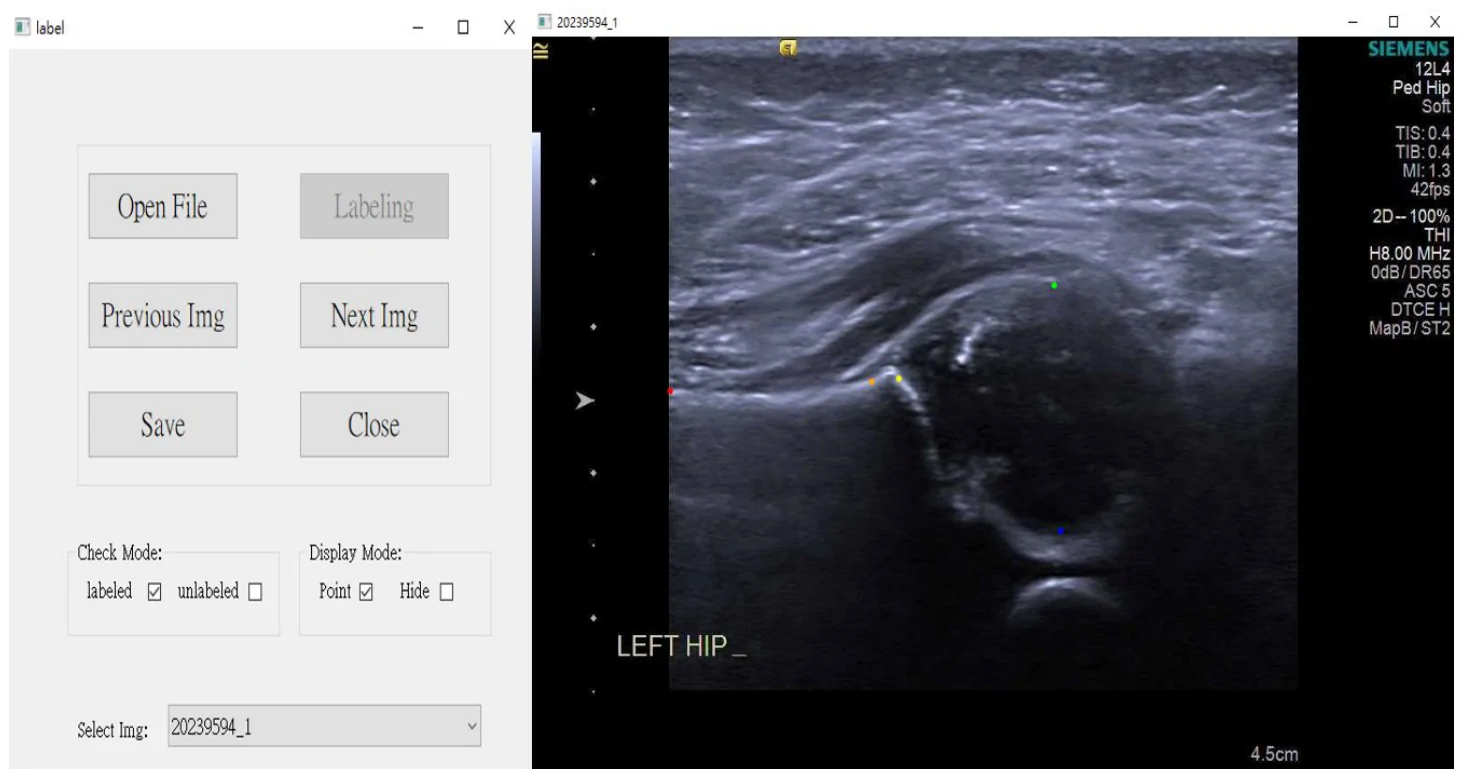
Key point annotation software.

The reference key points were determined jointly by two pediatric orthopedic surgeons certified in infant hip ultrasonography (C.-A.S. and C.-K.H.) using a custom annotation tool (Figure 5). The annotators were blinded to clinical information, and any disagreement was resolved through discussion with a senior orthopedic surgeon (C.-J.L.) until consensus was reached.

### 2.2. Data Partitioning and Implementation

The model was implemented using PyTorch framework, running on the Ubuntu 20.04 operating system. The hardware setup includes an Intel^(R)^ Core^(TM)^ i7-13700K CPU @ 5.40 GHz and a NVIDIA GeForce RTX 4090 with 24 GB of memory. The entire ultrasound image data set was divided into training sets and testing sets in a 4:1 ratio by using fivefold cross validation, with an original image resolution of 1024 × 768 pixels. We trained the model for 300 epochs with a batch size of 4, using the RMSprop optimizer, a learning rate of 0.0002, and α set to 0.8. During the training process, we employed fivefold cross-validation to evaluate the generalization performance by training on different segments of the data set. Each fold’s training set contained 240 pediatric hip ultrasound images, and the testing set included 60 pediatric hip ultrasound images. The ratio of number of normal and abnormal images was also 2:1. No one hip image was simultaneously deployed in training and test data sets when the fivefold cross validation was used. Fivefold cross-validation was performed at the hip level: each hip contributed a single image, so that no hip appeared in both the training and test sets of any fold, whereas the right and left hips of the 135 infants with bilateral images could be allocated to different folds.

### 2.3. Key Point Detection Model

**Figure 6:**
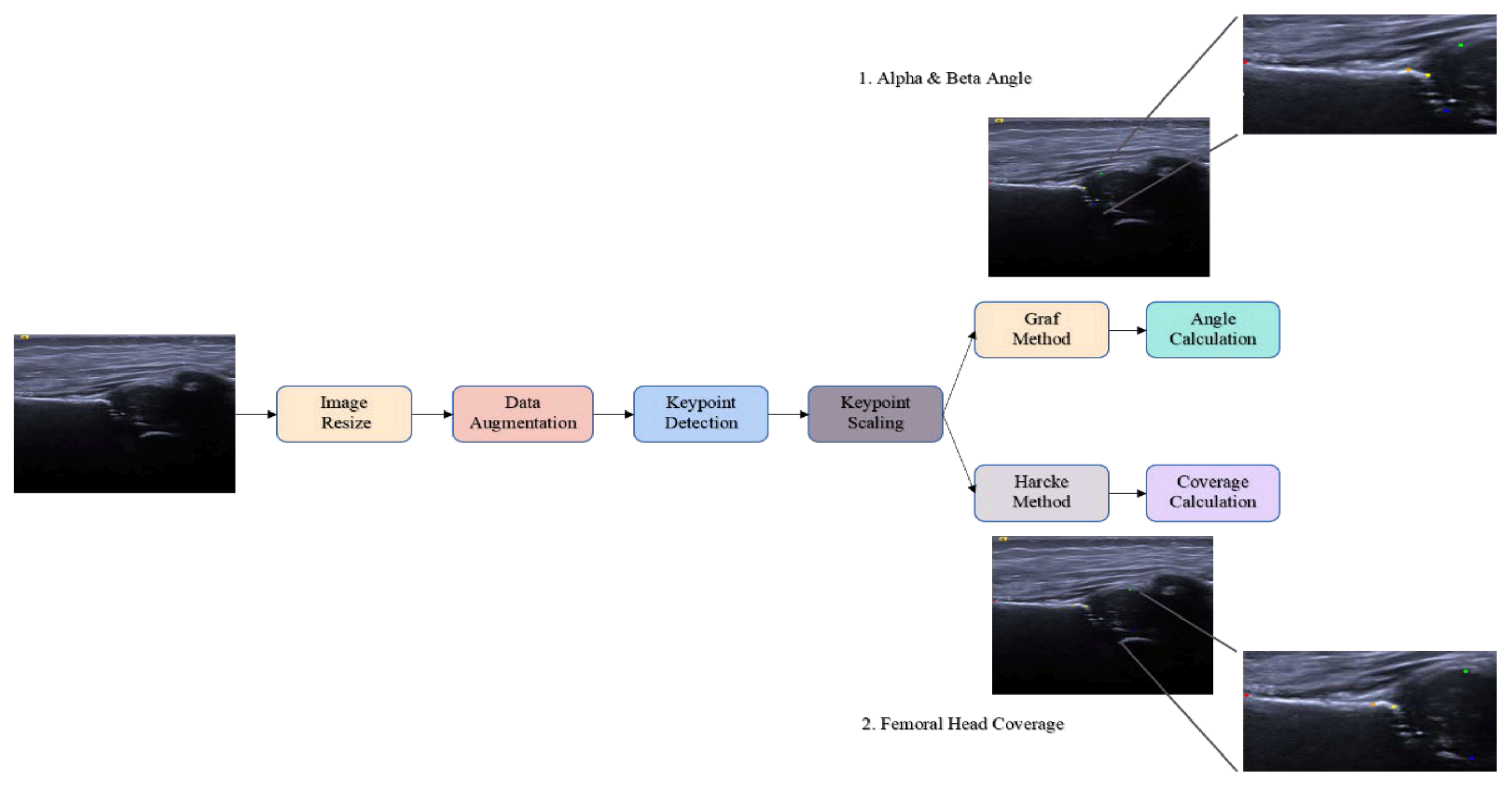
Flowchart of DDH-KPNet model.

In this study, we designed an automated system for processing hip ultrasound images, integrating key point detection techniques from computer vision and utilizing deep learning algorithms. This system predicted crucial key points on the hip joint and assessed hip conditions using different evaluation methods, such as Graf’s method and Harcke’s method, to assist orthopedic doctors in clinical diagnosis. When evaluating pediatric hip dysplasia, orthopedic doctors measure the angles and the coverage to assess the severity of the condition. Our goal was to automatically detect the necessary key points through deep learning methods. In Figure 6, the system workflow for detecting key points in pediatric hip ultrasound images is shown. The detection targets were divided into two categories: α and β angles, and femoral head coverage. First, all ultrasound images were resized to 256 × 256 pixels (without any padding and cropping) and subjected to a series of data augmentation operations such as geometric transformations and contrast adjustment. Then, a key point detection network was utilized to predict the positions of five key points based on the detection target. Finally, by obtaining the positional information of the key points, we accurately mapped each key point back to its corresponding position on the original image to further analyze the angles and coverage formed by the key points.

#### 2.3.1. CNN Branch

In the original transfuse architecture, the authors introduced the ResNet [25] architecture into the CNN branch, constructing a deep network through multiple stacked residual network modules and incorporating skip connections to directly pass shallow layer input feature information to subsequent and deeper layers.

Our proposed method utilizes an improved architecture based on Unet++ as the backbone of the CNN branch. Research has demonstrated that Unet excels in medical image feature extraction, and Unet++ introduces additional skip connections and multiresolution feature fusion on top of the original Unet. The core idea was to add a series of dense convolutional layers between the encoder and decoder, with dense skip connections between these layers. This setup allows the encoder, or backbone, to perform a series of downsampling and convolution operations to extract deep feature information. The upsampling and skip connections then link features of different resolutions, improving feature fusion and propagation. Ultimately, the multiresolution features obtained from the decoder are fused with those from the transformer branch.

**Figure 7:**
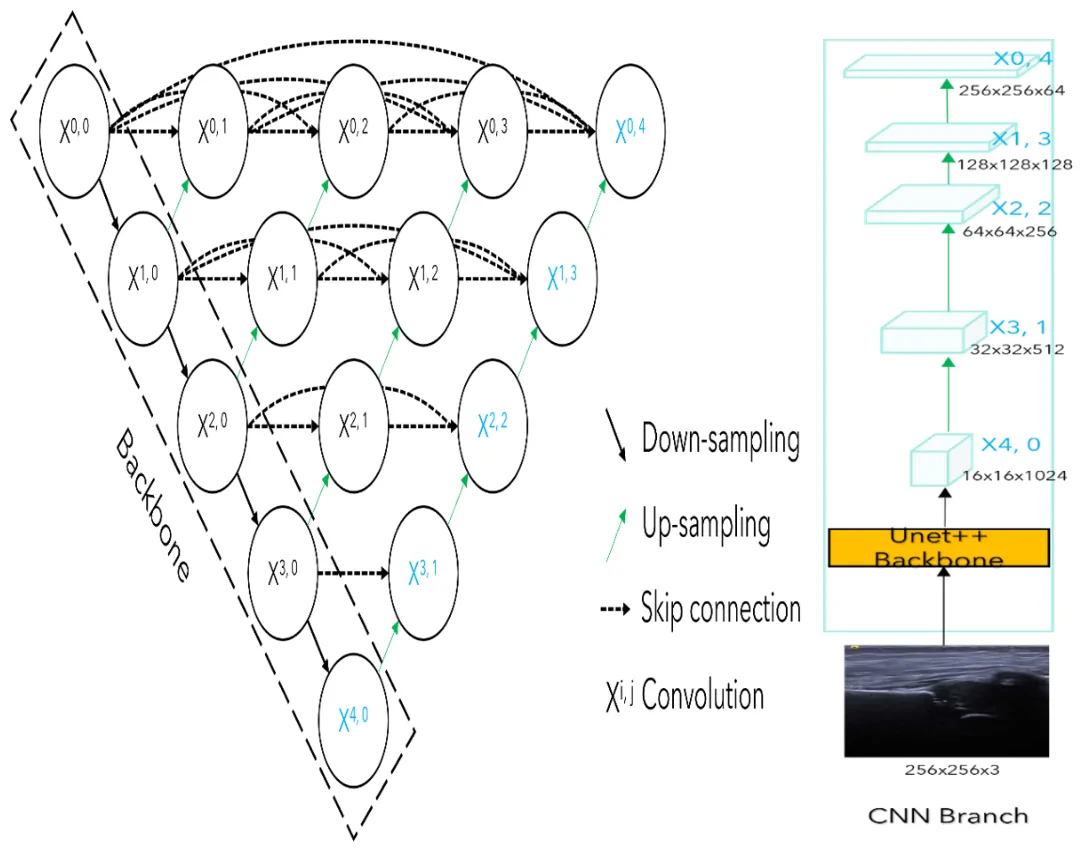
Convolutional neural network branch employs the Unet++ model.

In our convolutional neural network branch, the aggregated feature vectors extracted by the Unet++ backbone are utilized during the upsampling stage. The multiscale feature maps obtained through convolutions X^0.4^, X^3.1^, X^2.2^ in the model are fused with the output from the transformer branch. Additionally, during the final upsampling stage of Unet++, a high-resolution feature map is obtained after convolution X^0.4^, which outputs spatial detail information to the prediction head. This feature map is then concatenated with the features from the transformer branch and the attention gate-fused features to retain more detail, serving as the heatmap for the model’s predictions. Figure 7 shows the convolutional neural network branch using the decoder of Unet++ model.

#### 2.3.2. Transformer Attention Mechanism Branch

**Figure 8:**
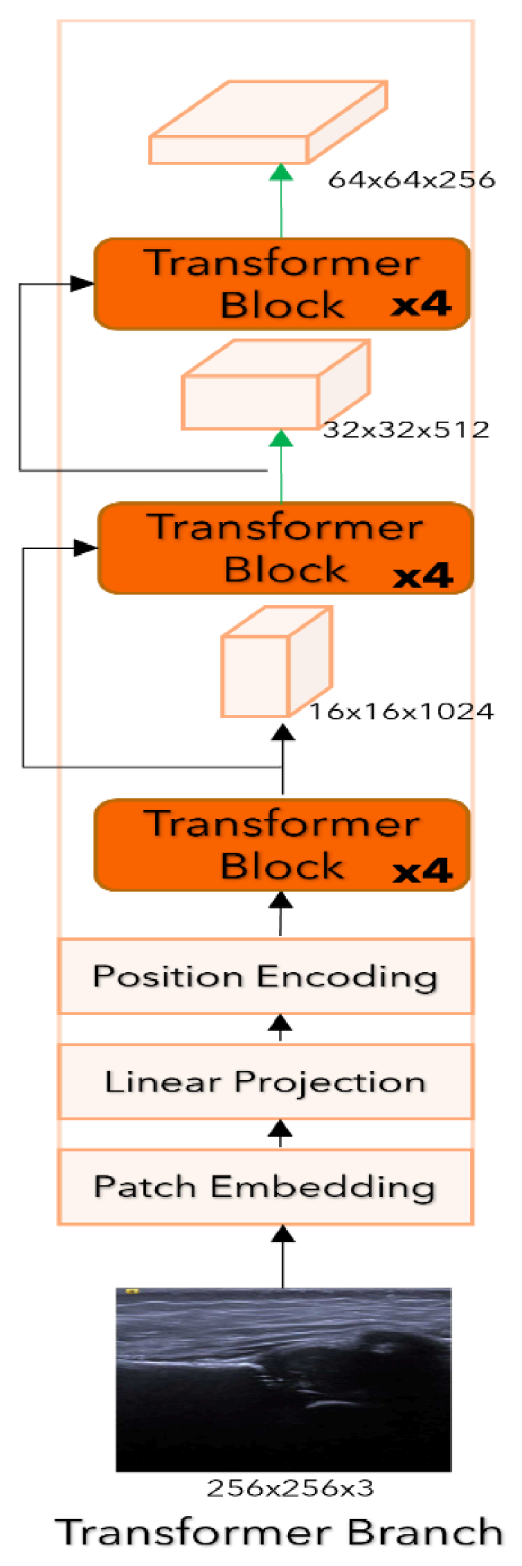
Revised vision transformer model.

The original transfuse architecture used the vision transformer [26] framework as the transformer attention mechanism branch to achieve global perception through attention mechanisms. Originally, the vision transformer outputs three different scales of feature maps after passing through 12 transformer blocks and upsampling. These feature maps are used for fusion with multiple feature maps from the convolutional neural network branch. Our proposed approach modified the vision transformer structure, as shown in Figure 8. We now output a feature map at each scale after every four layers of transformer module operations. This modification allows our transformer structure to learn shallow and deep feature details. The hierarchical concept in our adjusted structure has also demonstrated improved performance in subsequent experiments.

#### 2.3.3. Feature Fusion Module

**Figure 9:**
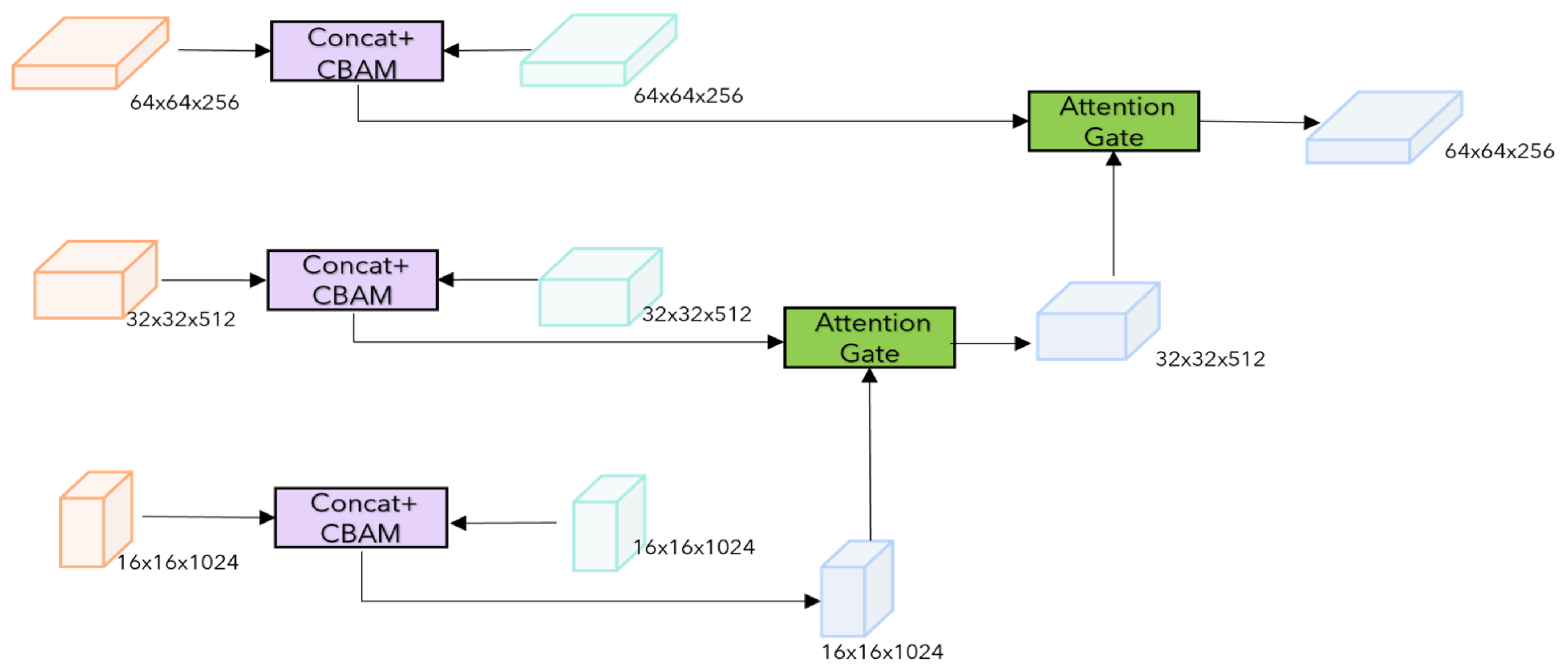
Feature Fusion Module architecture.

In our key point detection model, after obtaining different scale features from the CNN branch and the transformer branch, as shown in Figure 9, we concatenated the feature maps from both branches and introduced the convolutional block attention module (CBAM) [27] mechanism. This approach allows the model to fuse the most relevant information in the channel and spatial directions, enhancing the input features and potentially improving the model’s interpretability and performance. After fusing the features from both branches, we used attention gate (AG) [28] mechanisms to aggregate the feature maps at different scales. This method selectively enhances low-level features while suppressing irrelevant regions, thus improving cross-layer feature fusion.

#### 2.3.4. Prediction Head

**Figure 10:**
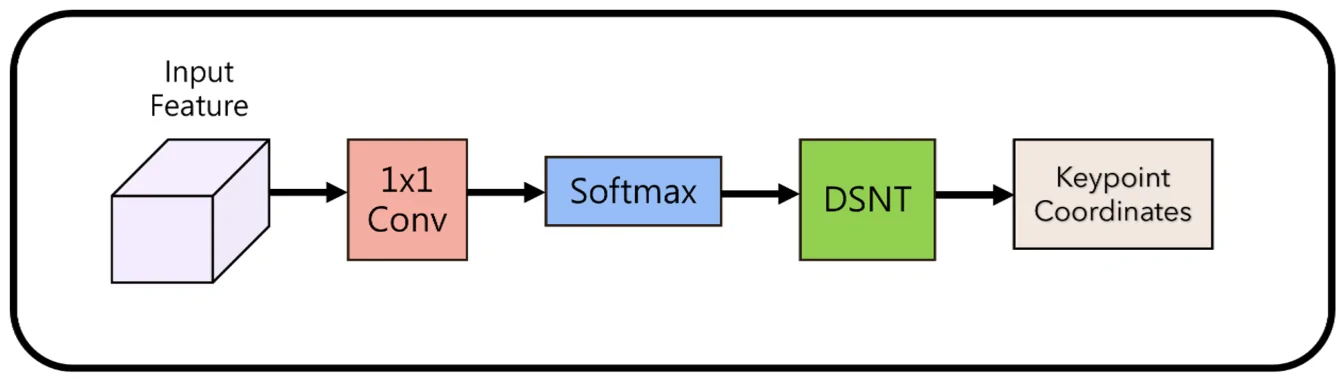
Prediction head architecture.

Figure 10 illustrates the process of predicting key points using features obtained from the CNN branch, the transformer branch, and the multiscale features fused through the attention gate. First, the feature maps are converted into heatmaps of size *H* × *W* × C using a 1 × 1 convolution layer, where C represents the number of key points to be predicted. After applying the Softmax layer, we obtained a probability distribution map based on the heatmap. Subsequently, the differentiable spatial to numerical transform (DSNT) [29] was applied to convert the heatmaps into numerical key point coordinates.

#### 2.3.5. Loss Function

In our approach, we used the root mean square distance (rmsd) loss to compute the loss between the ground truth key points *d_i_* = (*x_i_*, *y_i_*) and the predicted key points ${\hat{d}}_{i}=({\hat{x}}_{i}$, ${\hat{y}}_{i})$, as shown in Equation (2). Here, n denotes the number of the pair of the *d_i_* and ${\hat{d}}_{i}$ key points. The rmsd loss was used to construct the core component of our loss function.

(2) $$L_{\mathrm{rmsd}}=\sqrt{\frac{1}{n}\sum_{i=1}^{n}[{\left(x_{i}-{\hat{x}}_{i}\right)}^{2}+{\left(y_{i}-{\hat{y}}_{i}\right)}^{2}]}$$

To ensure that the predicted heatmaps gradually resemble Gaussian distributions centered around the key point coordinates, the ground truth heatmaps were generated using Gaussian kernels. These ground truth heatmaps were then compared with the model’s predicted heatmaps to compute the error in the loss function. The Gaussian kernel is defined as shown in Equation (3), where (*i_n_*, *j_n_*) denotes the coordinates of the *n*th 2D key point, and *σ* represents the standard deviation of the Gaussian kernel, which is set to 2.

(3) $$H_{n}\left(i,\;j\right)=\exp(-\frac{{\left(i-i_{n}\right)}^{2}+{\left(j-j_{n}\right)}^{2}}{2\sigma^{2}})$$

We applied strict regularization to the appearance of the heatmaps to achieve better data fitting through faster convergence. We used the Jensen–Shannon divergence loss to measure the difference between two distributions, as shown in Equation (4). Here, *JSD*(·∥·), *Heatmap_pred_*, and *Heatmap_gt_* represent the Jensen–Shannon divergence measure, the predicted heatmap, and the ground truth heatmap, respectively.

*L_D_* = *JSD*(*Heatmap_pred_* ∥* Heatmap_gt_*) (4)

The mathematical expression for Jensen–Shannon divergence is given in Equation (5). Here, *JSD* represents the Jensen–Shannon divergence, where *P* is *Heatmap_pred_*, *Q* is *Heatmap_gt_*, and *M* is defined as $\frac{1}{2}\left(P+Q\right)$. *KL*(X∥Y) denotes the Kullback–Leibler divergence, which is an asymmetric measure of the difference between two probability distributions *X* and *Y*. It is defined as $\sum_{i}X(i)\log\frac{X\left(i\right)}{Y\left(i\right)}$.

(5) $$\mathrm{JSD}\left(P∥Q\right)=\frac{1}{2}\;\mathrm{KL}\left(P∥M\right)+\frac{1}{2}\mathrm{KL}\left(Q∥M\right))$$

### 2.4. Statistical Analysis

Key point localization error was expressed as the mean absolute error (mm) between the predicted and reference coordinates and as the percentage of correct key points within 0.5 and 1.0 mm (PCK@0.5/1.0 mm). Agreement between reference and predicted continuous measurements (α angle, β angle, and femoral head coverage) was assessed using Bland–Altman analysis (mean bias and 95% limits of agreement) [30], the ICC (two-way random effects, absolute agreement, single measurement) [31], and the mean absolute error (MAE); Pearson’s *r* and *R*^2^ are reported as correlation and robustness measures. Diagnostic performance was reported as sensitivity, specificity, recall, precision, and accuracy, and the area under the receiver operating characteristic curve (AUC) was calculated using the predicted α angle and coverage as continuous scores.

## 3. Experimental Results

### 3.1. Key Point Detection

For key point detection of the α and β angles, we conducted fivefold cross-validation. As shown in Table 2, we calculated the mean absolute errors (MAEs) in coordinate positions between the physician-annotated ground truth and the model predictions. The cross-validation results yielded average deviations of 0.23 ± 0.26, 0.32 ± 0.36, 0.39 ± 0.29, 0.58 ± 0.39, and 0.41 ± 0.40. Further, the percentages of MAEs less than 0.5 and 1.0 mm are listed in Table 2. The deviations are measured in millimeters, with a conversion factor of 1 pixel = 0.071 mm. Point 1 exhibits the smallest deviation, indicating relatively better prediction at the bone boundary. Point 4 is located at the bony rim. Point 1, located on the straight iliac contour, exhibits the smallest deviation, reflecting the well-defined interface between bone and soft tissue at this location. Point 4 is located at the center of the acetabular labrum, which lies within the hypoechoic cartilaginous roof and has no sharply defined boundary. Table 2 reveals that the detection of Point 4 is worse than other points. The average MAE of five points is 0.368 mm.

As shown in Table 3, when comparing different feature extraction network methods, our method achieved the best key point prediction accuracies (mean ± standard deviation of mm), with results of 0.23 ± 0.26, 0.32 ± 0.36, 0.39 ± 0.29, 0.58 ± 0.39, and 0.41 ± 0.40, outperforming other methods. Methods like Unet++ and HRNet [32] performed better in prediction accuracy than the transformer-based HRFormer [33]. By utilizing our method, which combines features from transformers and convolutional neural networks, the model benefits from the global search capability of transformers. This allows it to capture fine spatial details in ultrasound images, enhancing the performance in predicting small key point positions.

### 3.2. Key Point Detection for Femoral Head Coverage

As shown in Table 4, for key point detection related to femoral head coverage, we conducted fivefold cross-validation. The average MAEs between the doctor-annotated ground truth and model predictions for key point coordinates were 0.26 ± 0.42, 0.53 ± 0.47, 0.54 ± 0.40, 0.72 ± 0.54, and 1.06 ± 0.90 measuring in millimeter distance. Point 4 and Point 5 correspond to the lateral (upper) and medial (lower) margins of the femoral head, respectively. Due to potential blurriness or shadowing in ultrasound imaging, these points exhibit greater variability in deviation. The average MAE of five points is 0.622 mm.

In order to analyze the influence of age variation to localize the key points, the three age intervals, one interval of age < 6 weeks, another interval between 6 weeks and 3 months, and the third interval of age > 3 months, are separately computed for MAE, as shown in Table 5. The result of Table 5 reveals that the key points prediction of older infants is more accurate.

As shown in Table 6, we compare different feature extraction network methods. The results indicate that our proposed method achieved key point prediction accuracies (mean ± standard deviation) of 0.26 ± 0.42, 0.53 ± 0.47, 0.54 ± 0.40, 0.72 ± 0.54, and 1.06 ± 0.90. The deviation for Point 5 is 1.06 ± 0.90, which is only slightly higher than the 1.03 ± 0.81 from HRNet, while the predictions for the other key points were the best overall. Our proposed method shows improved performance in identifying the contour and proportions of the femoral head, particularly for challenging key point locations like Point 4 and Point 5, resulting in better localization of the superior and inferior edges of the femoral head, particularly for the lateral margin of the femoral head (Point 4), whereas the error for the medial margin (Point 5) was comparable to that of the best comparison model.

### 3.3. Classification Performance

After measuring the α and β angles by the Graf method and femoral head coverage by the Harcke method, we categorized the results into normal and abnormal groups. For the α and β angles, a Type I classification signifies a normal hip joint, while Types IIa/IIb and IIc are identified as abnormal. Regarding femoral head coverage, a coverage rate exceeding 50% is deemed normal, whereas a rate below 50% is considered abnormal. The outcomes were analyzed using a confusion matrix, which classifies the results into true positive (TP), false positive (FP), true negative (TN), and false negative (FN). TP represents the number of instances where the actual and predicted values are true; FP denotes cases where the actual value is false, but the predicted value is true; TN corresponds to cases where the actual and predicted values are false, and FN refers to instances where the actual value is true, but the predicted value is false. The reference standard for classification was the Graf type and coverage category calculated from the expert-annotated key points using the same geometric rules applied to the predicted key points.

This study uses precision, recall, and accuracy as evaluation metrics, as listed in Equations (6)–(8), for our symptom classification. Recall represents the proportion of correctly predicted positive samples out of the total number of actual positive samples. Precision indicates the proportion of correctly predicted positive samples out of the total number of predicted positive samples. Accuracy measures the proportion of correctly predicted positive and negative samples out of all samples.

(6) $$\mathrm{Precision}=\frac{\mathrm{TP}}{\mathrm{TP}+\mathrm{FP}}$$

(7) $$\mathrm{Recall}=\frac{\mathrm{TP}}{\mathrm{TP}+\mathrm{FN}}$$

(8) $$\mathrm{Accuracy}=\frac{\mathrm{TP}+\mathrm{TN}}{\mathrm{TP}+\mathrm{FP}+\mathrm{TN}+\mathrm{FN}}$$

As shown in Table 7, when evaluating developmental dysplasia of the hip based on the α and β angles, and comparing different feature extraction methods, our proposed model achieved the best results in symptom classification with a precision of 89.0%, recall of 81.0%, and accuracy of 90.3%. The details of classification performance (under 95% CI) of DDH-KPNet are true positive rate 0.81, true negative rate 0.9497, false negative rate 0.19, false positive rate 0.0053, sensitivity 0.81, specificity 0.9497, and AUC 0.8799.

As shown in Table 8, when evaluating developmental dysplasia of the hip based on femoral head coverage and comparing different feature extraction methods for symptom classification accuracy, our proposed model architecture achieved a precision of 93.4%, which is slightly lower than 94.4% from HRNet. However, it achieved the best recall of 88.8% and accuracy of 94.2%. Details of classification performance of DDH-KPNet are true positive rate of 0.888, true negative rate of 0.968, false negative rate of 0.112, false positive rate of 0.0312, sensitivity of 0.888, specificity of 0.968 and AUC of 0.9284.

### 3.4. Correlation Analysis

The Pearson correlation coefficient is applied for this analysis. The Pearson correlation coefficient is a widely used metric for measuring the degree of linear relationship between two variables. To calculate *r*, we divide the covariance of the two variables by the product of their standard deviations, providing a standardized measure that facilitates comparison across different data sets. This method allows us to thoroughly assess the linear relationship between model predictions and annotated true values, thereby understanding the model’s performance and areas for improvement. The calculation method is shown in Equation (9), where *y_i_* represents the annotated true values, *y_i_* represents the model predictions, $\bar{y}$ is the mean of the annotated true values, ${\bar{\hat{y}}}_{i}$ is the mean of the model predictions, and *n* is the amount of data. The results of this correlation analysis will help identify the strengths and weaknesses of the model, providing a basis for future model improvements and optimization. A high correlation coefficient will enhance our confidence in the model’s accuracy and support its broader application in practice.

(9) $$r=\frac{\sum_{i=1}^{n}(y_{i}-\bar{y})({\hat{y}}_{i}-\bar{\hat{y}})}{\sqrt{{\sum_{i=1}^{n}(y_{i}-\bar{y})}^{2}}\sqrt{{\sum_{i=1}^{n}({\hat{y}}_{i}-{\bar{\hat{y}}}_{i})}^{2}}}$$

As shown in Table 9, we performed Pearson correlation analysis between physician-annotated true values and model predictions for the α and β angles and compared the results across other feature extraction methods. The results indicate that our method achieved the best correlation with *r* = 0.841 for the α angle and *r* = 0.734 for the β angle, demonstrating a high positive correlation between the model predictions and the annotated true values. Specifically, there are strong positive correlations for both the α angle and β angle.

Additionally, when comparing traditional convolutional neural networks such as Unet++ and HRNet with the transformer model HRFormer, the transformer model exhibited lower correlation results for α and β angles. This highlights that transformer models, due to their sensitivity to global information and their tendency to respond to more feature points at potential key point locations, exhibit greater variability in positional differences in predictions. In contrast, integrating the transformer with convolutional networks in our method improves performance by adding detailed local spatial information, thus reducing variability and increasing accuracy in β angle predictions.

In terms of femoral head coverage, as shown in Table 10, we compared the analysis results across different feature extraction methods. Our method achieved the best correlation with *r* = 0.939 for femoral head coverage, demonstrating an extremely high positive correlation between the model predictions and the annotated true values. After integrating the two foundational networks, our model better identifies the contour and proportion of the femoral head, resulting in more subtle changes in coverage prediction variability.

The coefficient of determination (*R*^2^) indicates how well the independent variable(s) explain the variation in the dependent variable. In other words, it shows the proportion of the total variation in the outcome that can be attributed to the model’s predictions. *R*^2^ ranges from 0 to 1. The calculation is given by Equation (10), where *y_i_* represents the annotated true values, ${\hat{y}}_{i}$ represents model predictions, $\bar{y}$ is the mean of the model predictions, and *n* is the amount of data. Negative *R*^2^ values can occur in some situations, particularly when the model’s fit is worse than a simple average model (where all predictions equal the mean of the observations). The value of the coefficient of determination depends not only on the complexity of the model, but also on the nature of and variability in the data.

(10) $$R^{2}=1-\frac{{\sum_{i=1}^{n}\;(y_{i}-{\hat{y}}_{i})}^{2}}{{\sum_{i=1}^{n}\;(y_{i}-\bar{y})}^{2}}$$

Table 11 shows the determination analysis between the physician-annotated true values and model predictions for the α and β angles and compares the results across different feature extraction methods. The results indicate that our method achieved the best coefficient of determination, with *R*^2^ = 0.709 for the α angle and *R*^2^ = 0.529 for the β angle. This demonstrates that the model’s predictions explain the variability in the true values better than other methods.

In terms of femoral head coverage, as shown in Table 12, we compared the analysis results across different feature extraction methods. Our method achieved the best coefficient of determination with *R*^2^ = 0.862. This indicates that, in terms of predicting femoral head coverage, the model’s predictions exhibit greater stability and less deviation compared with the α and β angles, showing a more consistent explanation of variability in the true values.

### 3.5. Agreement Analysis

**Figure 11:**
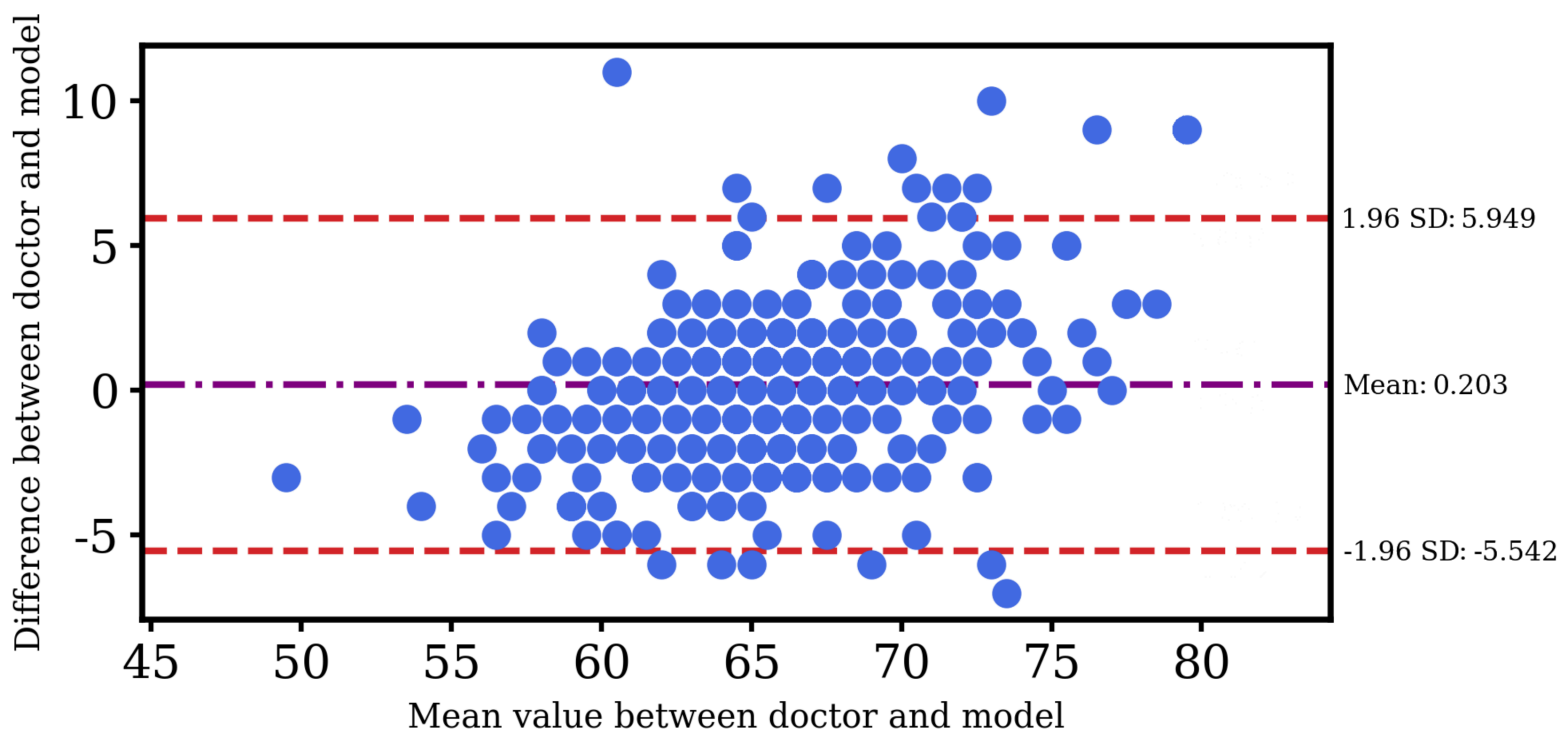
Bland–Altman plot of angle α (mean bias = 0.203, LOA = [−5.542, 5.949]).

**Figure 12:**
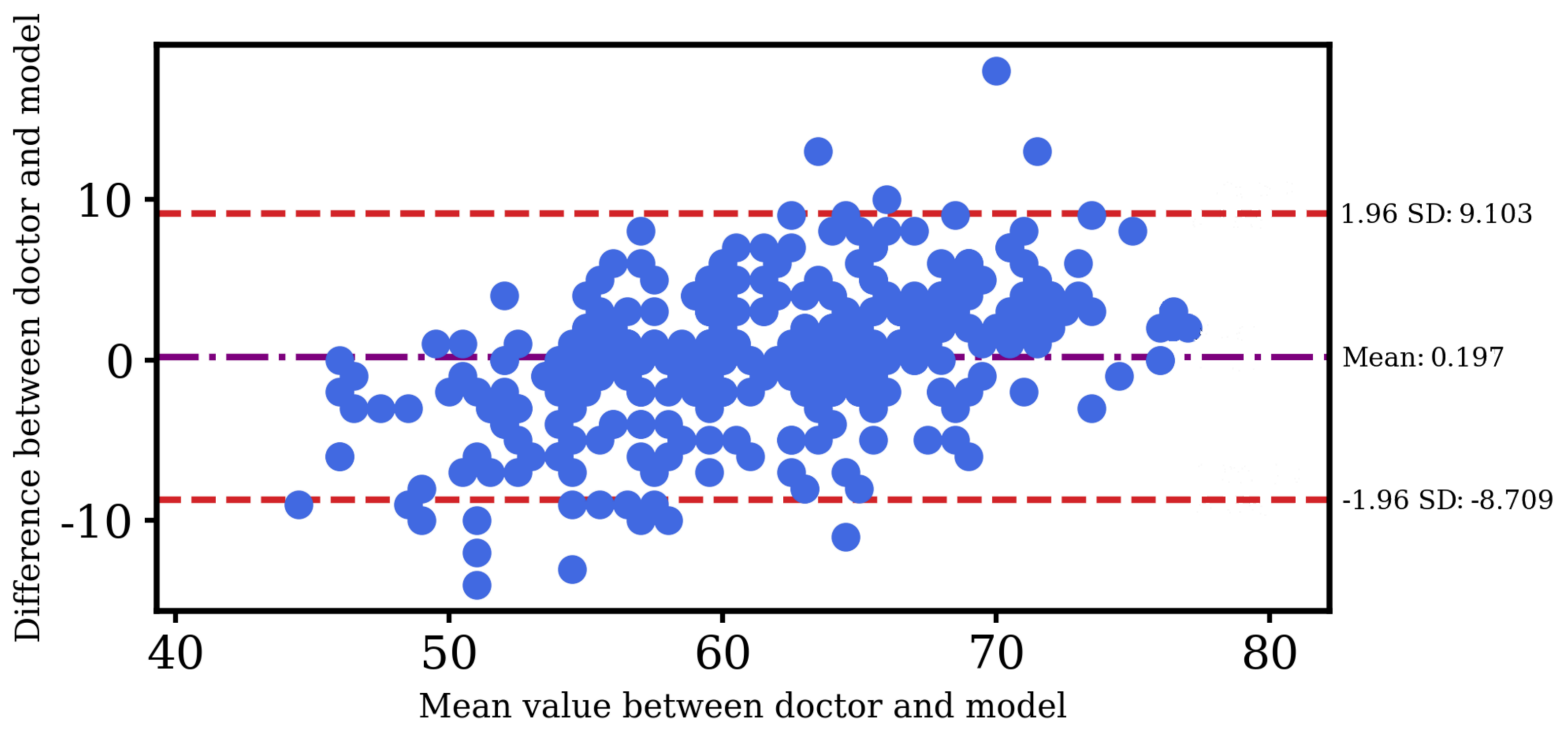
Bland–Altman plot of the *β* angle (mean bias = 0.197, LOA = [−8.709, 9.103]).

**Figure 13:**
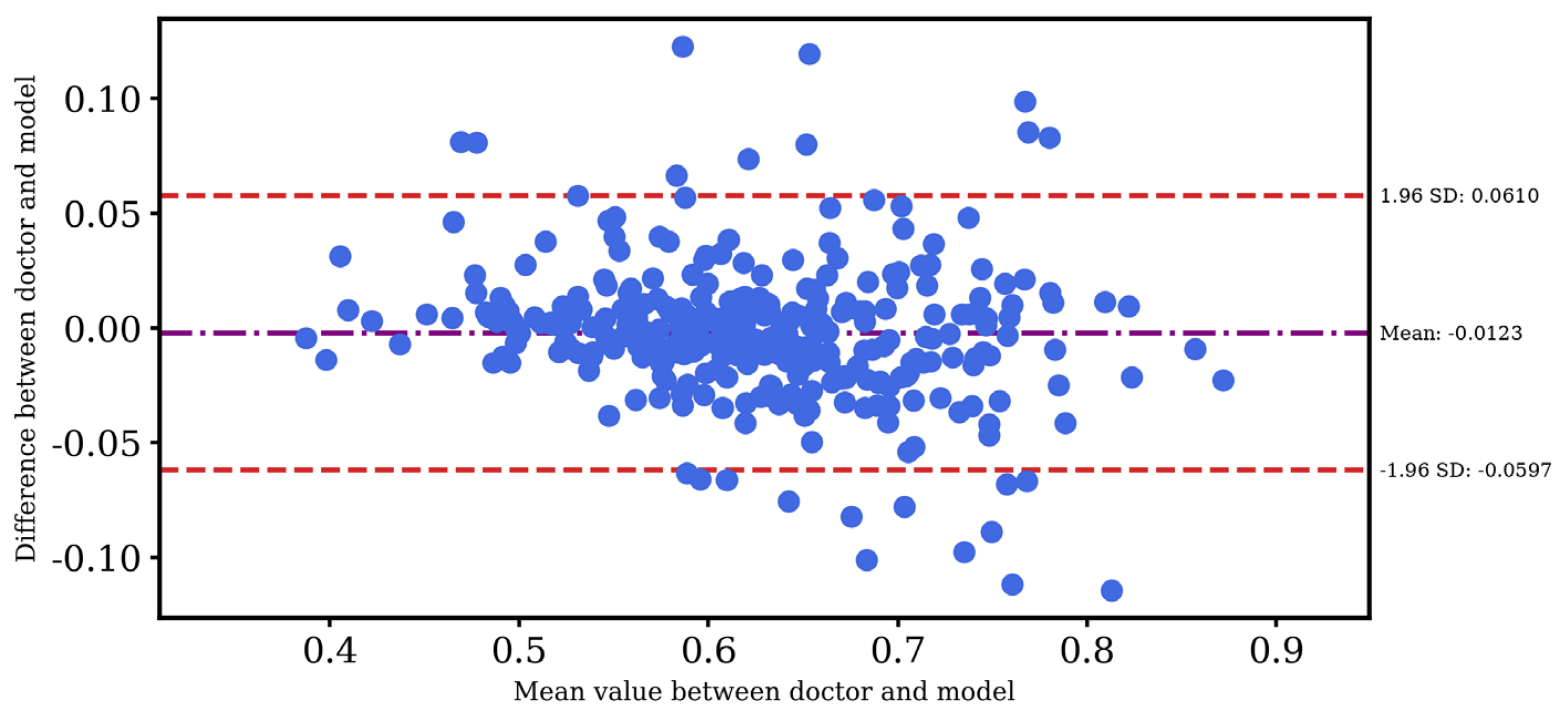
Coverage of Bland–Altman plot (mean bias = −0.0123, LOA = [−0.0597, 0.0610]).

In this study, as shown in Table 13, we used the paired Student’s *t*-test to evaluate the differences between the doctor-annotated values and the predicted values for the α angle, β angle, and femoral head coverage under 95% interval. This t-test compares the mean differences between paired samples (i.e., the true values and their corresponding model predictions) to determine if the differences are statistically significant. Specifically, the paired t-test is shown in Table 13, for measuring the mean differences between two related samples under different conditions. The experimental results show that, for the α angle, β angle, and femoral head coverage, our method yielded a *p*-value greater than 0.05, indicating insufficient evidence to reject the null hypothesis *H*_0_ (i.e., the mean of ground truth is equal to mean of corresponding predicted results). Furthermore, the intraclass correlation coefficient (ICC) and the Bland–Altman plots for the α angle, β angle, and coverage are as shown in Table 14 and Figure 11, Figure 12 and Figure 13. The used ICC denotes a two-way random effects model with absolute agreement for single measurement. The results reveal that the predicted results of DDH-KPNet had high agreement with doctor-annotated results.

### 3.6. Ablation Study

In our key point detection network, we compared the use of ResNet50 and Unet++ within the convolutional neural network branch to assess their impact on overall model prediction accuracy. The results indicate that Unet++ significantly improves prediction accuracy compared with ResNet50. This enhancement is attributed to the encoder–decoder structure of Unet++ and its ability to integrate multiscale feature maps, allowing the model to more precisely detect key point locations. For α and β angle measurements, as shown in Table 15, the deviations for the five key points are 0.23 ± 0.26, 0.32 ± 0.36, 0.39 ± 0.29, 0.58 ± 0.39, and 0.41 ± 0.40, all of which are the best achieved.

For the measurement of femoral head coverage, as shown in Table 16, the deviations for the five key points are 0.26 ± 0.42, 0.53 ± 0.47, 0.54 ± 0.40, 0.72 ± 0.54, and 1.06 ± 0.90, all of which are the best achieved. Combining the results from both measurements, we can see that Unet++, with its encoder–decoder structure and the ability to fuse multiscale feature maps, aggregates more useful feature details. Therefore, we have selected Unet++ as the feature extraction network for our convolutional neural network branch.

We also compare the impacts of different attention modules—BiFusion module, CBAM, and attention gate—on key point detection accuracy. We evaluate the effects of these modules on feature fusion between the convolutional neural network branch and the transformer branch, comparing the use of the BiFusion module and CBAM. Additionally, we assess the impacts of using attention gate versus CBAM in the multilevel fusion stage after merging features from both branches on overall model prediction accuracy.

For α and β angle measurements, as shown in Table 17, the deviations for the five key points are 0.23 ± 0.26, 0.32 ± 0.36, 0.39 ± 0.29, 0.58 ± 0.39, and 0.41 ± 0.40. All are the best results achieved except on Point 3, for which the combination of the BiFusion module and CBAM showed a slightly lower deviation (0.38 vs. 0.39 mm).

For the measurement of femoral head coverage, as shown in Table 18, the deviations for the five key points are 0.26 ± 0.42, 0.53 ± 0.47, 0.54 ± 0.40, 0.72 ± 0.54, and 1.06 ± 0.90. All are the best achieved except on Point 2, for which two other configurations showed slightly lower deviations (0.51 vs. 0.53 mm). Considering the overall results and weighing the accuracy of key point prediction, we have selected CBAM and attention gate as the attention modules in our model architecture. CBAM, with its channel and spatial attention features, demonstrates superior performance in capturing key point features compared with the more complex BiFusion module. Additionally, using the attention gate to fuse multiscale feature maps allows for more precise extraction of potential key point location information.

## 4. Discussion

In this study, we developed a key point-based deep learning model that combines CNN and transformer branches to measure the Graf α and β angles and femoral head coverage on infant hip ultrasound images. Under fivefold cross-validation, the model achieved MAE measures of key point localization 0.386 mm (measured *α* and *β* angles) and 0.622 mm (measured coverage); Graf-based classification accuracy 0.89, sensitivity 0.81, and specificity 0.903 (under 95% conference interval); Harcke classification accuracy 0.934, sensitivity 0.888, and specificity 0.942 (under 95% conference interval); and ICCs for α = 0.832, β = 0.728, and coverage = 0.923. The strengths of this study include a single framework that provides Graf- and Harcke-based assessments from the same image; separate evaluations of key point localization, measurement agreement, and classification; and reference key points determined by consensus of certified pediatric orthopedic surgeons. Unlike previous key point-based studies that focused on a single measurement method [16,17,21,22], our framework therefore allows the two most widely used sonographic assessments to be examined side by side.

The model performed better for femoral head coverage than for Graf classification. Because both assessments were derived from the same images, acquired with the same system and annotated by the same examiners, this difference is unlikely to be explained by an imaging technique or examiner experience; rather, it reflects the geometry of each measurement and the locations of the diagnostic thresholds. First, the α and β angles are defined by lines connecting closely spaced landmarks. Because the bony roof line is short, a small displacement of its endpoints produces a disproportionately large angular change; for example, a perpendicular displacement of 0.5 mm at one end of an 8 mm bony roof line changes the α angle by approximately 3.6°. The bony rim (Point 3) and the lower limb of the os ilium (Point 5), which define the bony roof line and thus the α angle, had mean localization errors of 0.39 and 0.41 mm, respectively (Table 2); errors of this magnitude can change the α angle by a few degrees. The largest error in the Graf task was observed at the center of the acetabular labrum (Point 4, 0.58 mm), which defines the cartilage roof line together with Point 3 and lies within the hypoechoic cartilaginous roof without a sharply defined boundary. In contrast, femoral head coverage is a ratio of distances relative to the whole femoral head diameter, so localization errors of similar magnitude result in proportionally smaller changes. Second, the Graf classification combines two angles, allowing errors to accumulate. Third, and probably most important, many infant hips cluster near the α = 60° threshold that separates Type I from Type II hips, where small measurement errors can change the category. This mirrors clinical practice, in which interobserver differences of several degrees in the α angle are well documented and are most consequential near classification thresholds [7,8,9,10].

The medial (lower) margin of the femoral head (Point 5) showed the largest localization error in the coverage task (see Table 4 and Table 6). Clinically, this is expected: the unossified femoral head is hypoechoic with a speckled internal pattern, and this margin lies adjacent to the triradiate cartilage and the ossified ischium, where acoustic shadowing and the absence of a distinct tissue interface make the boundary poorly defined; once the femoral head ossification center appears, its acoustic shadow can further obscure this margin. Expert examiners face the same difficulty in routine practice. The difference between our method and HRNet for Point 5 (1.06 vs. 1.03 mm) corresponds to approximately 0.4 pixels and is not clinically meaningful.

The reference standards for classification were the Graf type and coverage category calculated from the expert-annotated key points using the same geometric rules applied to the model’s predictions. The classification analysis therefore reflects the effect of key point localization error and does not represent an independent clinical diagnosis. Because expert measurements are themselves subject to interobserver variability, particularly in borderline hips near the α = 60° or 50% coverage thresholds [7,8,9,10], some apparent model errors in borderline hips may fall within the range of human measurement variability. As the reference key points in this study were established by consensus, interobserver agreement could not be quantified directly; the interobserver differences of several degrees in the α angle reported in previous studies [7,8,9,10] provide a benchmark for interpreting the model’s errors.

Hip morphology changes rapidly during the first months of life as the acetabular roof matures, and, in some infants, the femoral head ossification center begins to appear. These changes alter the echogenicity of the bony rim and femoral head margins and may therefore affect key point localization. In addition, the Graf distinction between Types IIa and IIb hips (α: 50–59°) depends on age (younger or older than 12 weeks), rather than on image features [23]. Because the model outputs the α angle, the subtype can be assigned downstream by combining the predicted angle with the infant’s age; IIa and IIb were therefore not treated as separate image-based classes. We also acknowledge that grouping physiologically immature Type IIa hips with pathological hips in the binary “abnormal” category is a measurement-based convention, rather than a treatment threshold, which should be considered when interpreting specificity.

From a clinical perspective, false-negative results are the most consequential errors, because a missed dysplastic hip may delay treatment beyond the period in which abduction bracing is most effective and increase the need for closed or open reduction later. In the revised analysis, abnormal hips were classified as normal. The proposed system is therefore intended to assist, not replace, expert interpretation. In practice, an “indeterminate zone” (e.g., predicted α of 55–65° or coverage of 45–55%) could be used to flag borderline hips for mandatory expert review, prioritizing sensitivity over specificity.

Several developments are needed before clinical use. First, multicenter data sets acquired with different ultrasound vendors, transducers, and examiners are required, together with domain adaptation or harmonization strategies, to ensure robustness across devices and protocols. Second, the training data should be enriched with severe (Type D, III, and IV) hips and with older infants, in whom femoral head ossification alters image appearance. Third, an automated standard-plane quality check should precede key point detection, so that nonstandard images are rejected, rather than measured. Fourth, integrating age into the output would allow full Graf subtyping (IIa vs. IIb). Fifth, uncertainty estimates for each key point could be used to flag unreliable measurements. Finally, the framework could be extended to dynamic or transverse views and to radiographic key points in older infants.

This study has several limitations. First, all images were obtained at a single tertiary center over a short period, using a single ultrasound system and transducer, and were acquired by a single examiner; model performance may differ with other populations, operators, imaging protocols, and ultrasound systems. Second, the data set was relatively small (300 images from 165 infants). Third, Graf Types D, III, and IV hips and nonstandard-plane or low-quality images were excluded by design; the model’s performance for decentered hips and for suboptimal images encountered in routine practice therefore remains unknown, and this selection may lead to overestimation of performance. Fourth, the hip was used as the unit of analysis; although the two hips of an infant frequently differ in developmental status, they share infant-level factors and the imaging conditions of the same examination, and this within-infant correlation may have affected the estimates. Fifth, the reference key points were established by consensus, rather than by independent annotations, so interobserver agreement could not be quantified in this data set, and expert annotations are themselves subject to variability, especially in borderline hips. Finally, the model was evaluated only by internal cross-validation. Independent multicenter external validation, preferably prospective, is required before clinical implementation.

## 5. Conclusions

In this study, we developed a key point-based deep learning model that combines CNN and transformer branches to automatically measure the Graf α and β angles and femoral head coverage on infant hip ultrasound images. Under fivefold internal cross-validation, the model achieved that Graf-based classification accuracy = 0.89, sensitivity = 0.81, and specificity = 0.903 (under 95% conference interval); and Harcke classification accuracy = 0.934, sensitivity = 0.888, and specificity = 0.942 (under 95% conference interval); and ICCs for α 0.832, β 0.728, and coverage 0.923. The results had good agreement with expert measurements. These results are promising but preliminary. Because the model was developed and internally validated on a relatively small data set from a single center and a single ultrasound system, independent multicenter external validation, preferably prospective, is required before its clinical utility can be established. If validated, such a system could support clinicians by providing reproducible measurements and flagging borderline hips for expert review.

## Figures and Tables

**Table 1:** Graf classification.

Graf Classification		A	Β
Type I	Mature	≥60°	-
Type IIa/IIb	Physiologically immature (IIa, <12 weeks)/delayed ossification (IIb, ≥12 weeks)	50–59°	-
Type IIc	Critical range	43–49°	≤77°

**Table 2:** Five-fold cross-validation of MAEs (mean ± standard deviation under 95% CI) between doctor annotations and predictions of DDH-KPNet (for Graf model).

	Point 1	Point 2	Point 3	Point 4	Point 5
Fold 1	0.19 ± 0.11	0.23 ± 0.15	0.29 ± 0.20	0.63 ± 0.39	0.37 ± 0.44
Fold 2	0.16 ± 0.09	0.29 ± 0.27	0.32 ± 0.21	0.51 ± 0.31	0.33 ± 0.31
Fold 3	0.19 ± 0.17	0.29 ± 0.28	0.43 ± 0.37	0.53 ± 0.34	0.38 ± 0.35
Fold 4	0.21 ± 0.17	0.41 ± 0.51	0.49 ± 0.30	0.70 ± 0.50	0.49 ± 0.54
Fold 5	0.40 ± 0.46	0.37 ± 0.46	0.40 ± 0.28	0.52 ± 0.34	0.46 ± 0.28
Average	0.23 ± 0.26	0.32 ± 0.36	0.39 ± 0.29	0.58 ± 0.39	0.41 ± 0.40
≤0.5 mm (%)	85.05	69.15	64.78	41.87	58.90
≤1.0 mm (%)	99.85	97.05	98.23	85.93	92.99

(measured in millimeters).

**Table 3:** Comparison of key point MAEs (mean ± standard deviation) between doctor annotations and predictions with other models.

Method	Point 1	Point 2	Point 3	Point 4	Point 5
Unet++	0.26 ± 0.47	0.34 ± 0.32	0.40 ± 0.31	0.60 ± 0.42	0.44 ± 0.49
HRNet	0.37 ± 0.51	0.47 ± 0.46	0.50 ± 0.41	0.74 ± 0.50	0.52 ± 0.49
HRFormer	0.41 ± 0.54	0.51 ± 0.45	0.51 ± 0.37	0.73 ± 0.47	0.58 ± 0.47
Transfuse	0.37 ± 0.91	0.34 ± 0.34	0.42 ± 0.29	0.62 ± 0.44	0.43 ± 0.44
DDH-KPNet	0.23 ± 0.26	0.32 ± 0.36	0.39 ± 0.29	0.58 ± 0.39	0.41 ± 0.40

(measured in millimeters).

**Table 4:** Five-fold cross-validation of MAEs (mean ± standard deviation) for femoral head coverage between doctor annotations and predictions (for Harcke model).

	Point 1	Point 2	Point 3	Point 4	Point 5
Fold 1	0.21 ± 0.13	0.50 ± 0.26	0.51 ± 0.30	0.86 ± 0.71	1.18 ± 1.07
Fold 2	0.16 ± 0.11	0.52 ± 0.41	0.54 ± 0.42	0.65 ± 0.52	0.90 ± 0.93
Fold 3	0.13 ± 0.09	0.51 ± 0.35	0.59 ± 0.42	0.69 ± 0.51	0.66 ± 0.56
Fold 4	0.16 ± 0.14	0.39 ± 0.30	0.44 ± 0.29	0.72 ± 0.44	1.16 ± 0.83
Fold 5	0.64 ± 0.81	0.75 ± 0.76	0.61 ± 0.50	0.67 ± 0.43	1.40 ± 0.83
Average	0.26 ± 0.42	0.53 ± 0.47	0.54 ± 0.40	0.72 ± 0.54	1.06 ± 0.90
≤0.5 mm (%)	71.61	47.46	46.02	33.51	26.71
≤1 mm (%)	96.09	84.13	87.49	69.15	47.34

**Table 5:** MAEs of three different age intervals.

Age	<6 Weeks	6 Weeks~3 Months	3~6 Months
number of infant hip	95	103	102
mean of MAE (Graf model)	0.412 mm	0.394 mm	0.264 mm
mean of MAE (Harcke model)	0.714 mm	0.674 mm	0.612 mm

**Table 6:** Comparison of key point MAEs (mean ± standard deviation of mm) for femoral head coverage between doctor annotations and predictions with other models.

Method	Point 1	Point 2	Point 3	Point 4	Point 5
Unet++	0.38 ± 1.04	0.57 ± 0.49	0.54 ± 0.40	0.73 ± 0.70	1.21 ± 1.37
HRNet	0.37 ± 0.51	0.63 ± 0.53	0.58 ± 0.43	0.88 ± 0.62	1.03 ± 0.81
HRFormer	0.39 ± 0.53	0.61 ± 0.50	0.58 ± 0.45	0.88 ± 0.61	1.06 ± 0.82
Transfuse	0.38 ± 0.78	0.55 ± 0.45	0.58 ± 0.40	0.81 ± 0.64	1.09 ± 0.94
DDH-KPNet	0.26 ± 0.42	0.53 ± 0.47	0.54 ± 0.40	0.72 ± 0.54	1.06 ± 0.90

**Table 7:** Comparison of symptom classification (precision, recall, and accuracy) by α and β angles between doctor annotations and predictions with other models.

Method	Precision	Recall	Accuracy
Unet++	88.0%	73.0%	87.7%
HRNet	82.4%	70.0%	85.0%
HRFormer	77.2%	61.0%	81.0%
Transfuse	81.0%	81.0%	87.0%
DDH-KPNet	89.0%	81.0%	90.3%

**Table 8:** Comparison of symptom classification (precision, recall, and accuracy) by femoral head coverage between doctor annotations and predictions with other models.

Method	Precision	Recall	Accuracy
Unet++	91.8%	83.8%	92.1%
HRNet	94.4%	83.8%	92.9%
HRFormer	90.5%	83.8%	91.7%
Transfuse	93.1%	83.8%	92.5%
DDH-KPNet	93.4%	88.8%	94.2%

**Table 9:** Pearson correlation analysis between doctor-annotated and predicted α and β compared with other models.

Method	*r*	
	*α*	β
Unet++	0.813	0.718
HRNet	0.717	0.563
HRFormer	0.672	0.574
Transfuse	0.621	0.656
DDH-KPNet	0.841	0.734

**Table 10:** Pearson correlation analysis between doctor-annotated and predicted femoral head coverage compared with other models.

Method	*r*
Unet++	0.918
HRNet	0.908
HRFormer	0.910
Transfuse	0.873
DDH-KPNet	0.939

**Table 11:** Coefficient of determination analysis between doctor-annotated and predicted α and β compared with other models.

Method	*R* ^2^	
	α	β
Unet++	0.654	0.510
HRNet	0.508	0.269
HRFormer	0.449	0.313
Transfuse	0.278	0.206
DDH-KPNet	0.709	0.529

**Table 12:** Coefficient of determination analysis between doctor-annotated and predicted femoral head coverage compared with other models.

Method	*R* ^2^
Unet++	0.810
HRNet	0.794
HRFormer	0.803
Transfuse	0.698
DDH-KPNet	0.862

**Table 13:** Student’s *t*-test between doctor-annotated and predicted α angle, β angle, and femoral head coverage (under 95% confidence interval).

A	β	Femoral Head Coverage
Accept null hypothesis *(p* = 0.311)	Accept null hypothesis (*p* = 0.949)	Accept null hypothesis (*p* = 0.526)

**Table 14:** The ICC of doctor-annotated and predicted α angle, β angle, and femoral head coverage (under 95% confidence interval).

	α	β	Coverage
**Intracorrelation coefficient (ICC)**	**0.832**	**0.728**	**0.923**

**Table 15:** Comparison of the impacts of Resnet and Unet++ on key point MAE (mean ± standard deviation of mm) for α and β angles between doctor annotations and predictions.

ResNet50	Unet++	Point 1	Point 2	Point 3	Point 4	Point 5
√		0.37 ± 0.91	0.34 ± 0.34	0.42 ± 0.29	0.62 ± 0.44	0.43 ± 0.44
	√	0.23 ± 0.26	0.32 ± 0.36	0.39 ± 0.29	0.58 ± 0.39	0.41 ± 0.40

**Table 16:** Comparison of the impacts of ResNet and Unet++ on key point MAE (mean ± standard deviation measuring in mm) for femoral head coverage between doctor annotations and predictions.

ResNet50	Unet++	Point 1	Point 2	Point 3	Point 4	Point 5
√		0.38 ± 0.78	0.55 ± 0.45	0.58 ± 0.40	0.81 ± 0.64	1.09 ± 0.94
	√	0.26 ± 0.42	0.53 ± 0.47	0.54 ± 0.40	0.72 ± 0.54	1.06 ± 0.90

**Table 17:** Comparison of the impacts of BiFusion module, CBAM, and attention gate on key point MAE (mean ± standard deviation measuring in mm) for α angle and β angle between doctor annotations and predictions.

BiFusion Module	Concat+ CBAM	Attention Gate	Point 1	Point 2	Point 3	Point 4	Point 5
√	√		0.25 ± 0.36	0.33 ± 0.35	0.38 ± 0.27	0.58 ± 0.41	0.42 ± 0.41
√		√	0.24 ± 0.26	0.34 ± 0.37	0.40 ± 0.28	0.61 ± 0.44	0.46 ± 0.46
	√		0.24 ± 0.30	0.33 ± 0.38	0.39 ± 0.29	0.62 ± 0.42	0.43 ± 0.42
	√	√	0.23 ± 0.26	0.32 ± 0.36	0.39 ± 0.29	0.58 ± 0.39	0.41 ± 0.40

**Table 18:** Comparison of the impacts of BiFusion module, CBAM, and attention gate on key point MAE (mean ± standard deviation measuring in mm) for femoral head coverage between doctor annotations and predictions.

BiFusion Module	Concat+ CBAM	Attention Gate	Point 1	Point 2	Point 3	Point 4	Point 5
√	√		0.27 ± 0.43	0.51 ± 0.45	0.55 ± 0.40	0.76 ± 0.53	1.11 ± 1.04
√		√	0.32 ± 0.60	0.54 ± 0.46	0.55 ± 0.39	0.77 ± 0.74	1.15 ± 1.28
	√		0.31 ± 0.49	0.51 ± 0.44	0.54 ± 0.40	0.75 ± 0.57	1.10 ± 1.00
	√	√	0.26 ± 0.42	0.53 ± 0.47	0.54 ± 0.40	0.72 ± 0.54	1.06 ± 0.90

## Data Availability

The data presented in this study are available on request from the corresponding author because of patient privacy restrictions.
